# Early atherosclerosis in normotensive patients with autosomal dominant polycystic kidney disease: the relation between epicardial adipose tissue thickness and carotid intima-media thickness

**DOI:** 10.1186/s40064-016-1871-8

**Published:** 2016-02-29

**Authors:** Saim Sag, Abdulmecit Yildiz, Sumeyye Gullulu, Fatih Gungoren, Bulent Ozdemir, Ercan Cegilli, Aysegul Oruc, Alparslan Ersoy, Mustafa Gullulu

**Affiliations:** Department of Cardiology, Uludag University Faculty of Medicine, Bursa, Turkey; Department of Nephrology, Uludag University Faculty of Medicine, Bursa, Turkey

**Keywords:** Autosomal dominant polycystic kidney disease, Preserved renal function, Early atherosclerosis, Epicardial adipose tissue thickness, Carotid intima-media thickness

## Abstract

Epicardial adipose tissue thickness (EATT) is suggested as a novel marker of subclinical atherosclerosis. Despite increased carotid intima-media thickness (CIMT) in autosomal dominant polycystic kidney disease (ADPKD) patients, the extent of the relationship between CIMT and EATT is unknown. The main purpose of our study was to evaluate the relation between EATT and CIMT in normotensive ADPKD patients with well-preserved renal function. Fifty-five normotensive ADPKD patients with normal renal function and 50 healthy control subjects were included in the study. EATT and CIMT were measured by echocardiography in all subjects. Correlation between EATT and CIMT was evaluated in ADPKD patients, while multivariate linear regression analysis was performed to determine factors predicting EATT and CIMT. ADPKD patients had significantly higher levels CIMT [0.7 (0.4–1.2) vs. 0.5 (0.4–0.8) mm, p < 0.001] and EATT (6.8 ± 2.7 vs. 4.8 ± 1.2 mm, p < 0.001) as compared with control subjects. Significant positive correlation was found between EATT and CIMT (r = 0.58, p < 0.001). Higher CRP levels (OR 54.7, 95 % CI 37.44–72.01, p < 0.001) and having ADPKD (OR 10.2, 95 % CI 2.53–17.86, p = 0.01) were the only independent factors associated with a higher EATT. A higher age (OR 0.35, 95 % CI −0.02 to 0.71, p = 0.06) tended to be independently associated with a higher EATT. In conclusion, our findings suggest that EATT, being simply measured by echocardiography and correlated with CIMT, can be used to detect subclinical atherosclerosis in normotensive ADPKD patients.

## Background

Autosomal dominant polycystic kidney disease (ADPKD), one of the most common hereditary diseases, is an important cause of end-stage renal disease (ESRD) (Schrier 2006; Ecder and Schrier 2009), while frequently associated with cardiovascular diseases as the leading cause of morbidity and mortality in ADPKD patients (Ecder and Schrier 2004, 2009; Grantham 2008).

Endothelial dysfunction (ED) is an early and reversible manifestation of atherosclerosis with multifactorial etiology. Arterial stiffness with increased vascular smooth muscle tone and intima media thickness (IMT) is related to ED (Widlansky et al. 2003; Sag et al. 2015; Turkmen et al. 2008; Yildiz et al. 2014). Given that ED develops in both normotensive and hypertensive ADPKD patients preceding the beginning of renal failure (Peterson et al. 2013), atherosclerosis has been considered to play a key role in the early phases of cardiovascular injury identified in the pathogenesis of ADPKD (Schrier 2006; Turkmen et al. 2008; Yildiz et al. 2014).

In this regard, identification of the mechanisms responsible for ED becomes important given the likelihood of early treatment and thus improved cardiovascular prognosis among affected ADPKD patients (Ecder and Schrier 2009; Peterson et al. 2013). Besides ED, other markers including LVH and carotid artery IMT (CIMT) have been used in past studies with normotensive ADPKD to show subclinical organ damage (Turkmen et al. 2008; Martinez-Vea et al. 2000; Kocyigit et al. 2014).

CIMT is a well-known independent predictor of cardiovascular diseases and recognized as a novel marker of subclinical atherosclerosis (Polak et al. 2011). In addition, on the basis of its strong correlation with obesity, insulin resistance, metabolic syndrome, hypertension, diabetes, and subclinical atherosclerosis (Polak et al. 2011; Iacobellis and Willens 2009); echocardiographic measurement of epicardial adipose tissue thickness (EATT) has also been indicated to be a potential marker of atherosclerosis and of CIMT and thus might be used as a simple tool for predicting cardiometabolic risk (Polak et al. 2011; Iacobellis and Willens 2009; Altun et al. 2014; Chaowalit et al. 2006).

While EATT is positively correlated with CIMT in several diseases (Iacobellis and Willens 2009; Altun et al. 2014; Natale et al. 2009; Kim et al. 2013), to our knowledge no data are available on the potential role of EATT in predicting early atherosclerosis as well as CIMT in patients with ADPKD. Therefore, our study was designed to evaluate the relationship between echocardiographic EATT and CIMT, as predictors of early atherosclerosis, in normotensive ADPKD patients.

## Methods

### Study population

Between July 2013 and July 2015, 115 ADPKD patients who were registered by the Uludag University School of Medicine in the Turkish Nephrology Society Cystic Kidney Disease Working Group Registry were evaluated for the study. Fifty-five normotensive ADPKD patients with normal renal function (mean ± SD age 38 ± 11.4 years, 26 males, 29 females) and 45 age- and sex-matched healthy subjects (mean ± SD age 38.6 ± 10.1 years, 24 males, 26 females) were included in the study. ADPKD was diagnosed and defined according to the ultrasonographic criteria published by Pei et al. (2009) and all patients had a positive family history of ADPKD. Presence of impaired renal function (estimated GFR, <60 mL/min/1.73 m^2^), hyperlipidemia, hypertension, or any cardiovascular disease, a measured office blood pressure ≥140/90 mmHg and usage of antihypertensive drugs were the exclusion criteria. After a detailed explanation each patient signed an informed consent form in accordance with the declaration of Helsinki. The local ethics committee of Uludag University approved the study.

### Study parameters

Data on patient demographics, anthropometrics [weight (kg), height (cm), body mass index (BMI; kg/m^2^)], vital signs [systolic blood pressure (mmHg), diastolic blood pressure (mmHg), pulse (bpm)], blood biochemistry [BUN (mg/dL), creatinine (mg/dL), uric acid (mg/dL), estimated glomerular filtration rate (GFR; mL/min), total cholesterol (mg/dL), HDL cholesterol (mg/dL), LDL cholesterol (mg/dL), triglycerides (mg/dL), uric acid (mg/dL) and high-sensitivity C-reactive protein (hs-CRP; mg/dL)], left ventricul ejection fraction (LVEF; %), left ventricular mass (LVM; g), CIMT (mm) and EATT (mm) were recorded in patient and control groups. Correlation between EATT and CIMT was evaluated in ADPKD patients, while multivariate linear regression analysis was performed to determine factors predicting EATT and CIMT.

### Measurements

After an overnight fasting venous blood samples were taken for biochemical analysis. Blood samples were analyzed for plasma glucose, BUN, creatinine, uric acid, total cholesterol, LDL-cholesterol, HDL-cholesterol and triglycerides using an autoanalyzer (Aeroset System Operations Manual; Abbot Laboratories, Abbott Park, IL, USA). The solid phase enzyme linked immunosorbent assay (ELISA) method was used with a High Sensitivity CRP Enzyme Immunoassay (DRG International Inc., Mountainside, NJ, USA) for the measurement of high-sensitivity C-reactive protein (hs-CRP). Estimated GFR was calculated based on the Chronic Kidney Disease Epidemiology Collaboration (CKD-EPI) equation (Levey et al. 2009).

### Echocardiography investigation

Two-dimensional (2D) transthoracic echocardiography was performed with a widely available transducer and equipment (M3S probe, Vivid 7, GE-Vingmed, Horten, Norway), by standard techniques, with subjects at rest, in the left lateral decubitus position. The EATT measurements were performed as previously defined by Iacobellis et al. (2003). The relatively echo-free space between the myocardial outer wall and the visceral pericardium on the anterior wall of the right ventricle at end-systole in the parasternal long-axis view was defined as EATT. The LVEF and LVM were calculated according to American Society of Echocardiography Guidelines (Lang et al. 2005).

CIMT was measured from 10 mm proximal to the right common carotid artery bifurcation segment, using the same device with a 12 MHz linear-array imaging probe. Carotid IMT was calculated as the distance between the lumen–intima and media–adventitia interfaces. Analysis was based on average of three consecutive measurements. The same expert cardiologist, unaware of the clinical data, performed and calculated EATT and CIMT. Intra-observer reproducibility was determined using a Spearman correlation coefficient. In our echo laboratory, the intra-observer correlation coefficients were 0.92 for CIMT and 0.91 for EATT, representing good reproducibility and reliability.

### Statistical analysis

Normality of distribution of the data was analyzed using Kolmogorov–Smirnov test. Categorical data were presented as numbers and percentages and were compared by Chi square test. Continuous data were presented as median (interquartile range) or mean ± standard deviations according to normality of distribution. Comparison of continuous data was performed using Mann–Whitney U or Student t tests as needed. Receiver operator characteristics curve was performed to define a cutoff level for EATT with optimal sensitivity and specificity to distinguish patients with ADPKD and controls. Spearman or Pearson tests were used for correlation analyses. Variables with a significant correlation with EATT in the univariate models were included in multivariate linear regression analysis to determine independent correlates of EATT. Among BP measurements, only strongest correlate of EATT was included in the model to avoid multicolinearity. A correlation coefficient of 0.1–0.3 was considered as weak, 0.3–0.5 as moderate, and >0.5 as strong correlation. A two-sided p value <0.05 was considered as statistically significant.

## Results

### Demographic, anthropometric and vital characteristics in study groups

Patient and control groups were similar in terms of age and gender distribution (Table 1). ADPKD patients had similar BMI, smoking rate and BP levels when compared with control subjects.

**Table 1:** Clinical and laboratory characteristics of ADPKD patients and control subjects

	ADPKD patients (n = 55)	Controls (n = 50)	p value
Age (years)	38 ± 11.4	38.6 ± 10.1	0.674
Gender (males/females)	26/29	24/26	0.548
Body mass index (kg/m^2^)	24.1 ± 3.4	23.1 ± 2.9	0.327
Smoking (%)	34	34.5	0.559
Systolic blood pressure (mmHg)^a^	123.2 ± 10.1	122.9 ± 7.4	0.873
Diastolic blood pressure (mmHg)^a^	75.7 ± 9.1	76.8 ± 6.8	0.499
BUN (mg/dL)	12 (8–28)	11 (7–24)	0.176
Creatinine (mg/dL)	0.76 ± 0.16	0.75 ± 0.11	0.881
Estimated GFR (mL/min)	107 ± 17	111 ± 17	0.270
Total cholesterol (mg/dL)	180 ± 28	179 ± 24	0.352
HDL cholesterol (mg/dL)	43.8 ± 8.4	45 ± 9.3	0.569
LDL cholesterol (mg/dL)	106 ± 28	104 ± 30	0.404
Triglycerides (mg/dL)	114 (44–700)	115 (43–333)	0.985
Uric acid (mg/dL)	4.8 ± 1.5	3.2 ± 0.9	<*0.001*
hs-CRP (mg/dL)	0.42 (0.22–1.19)	0.31 (0.13–0.9)	<*0.001*
Left ventricul ejection fraction (%)	72 (60–82)	70 (65–82)	*0.009*
Left ventricular mass (g)	188 ± 55	154 ± 36	<*0.001*
CIMT (mm)	0.7 (0.4–1.2)	0.5 (0.4–0.8)	<*0.001*
EAT thickness (mm)	6.8 ± 2.7	4.8 ± 1.2	<*0.001*

*ADPKD* autosomal dominant polycystic kidney disease, *GFR* glomerular filtration rate, *CIMT* carotid intima-media thickness, *EAT* epicardial adipose tissue, *hs*-*CRP* high sensitive C-reactive protein, *HDL* high density lipoprotein, *LDL* Low density lipoprotein

^a^Blood pressure measurements were performed at the office

The results in italics identify the statistically significant values

### Blood biochemistry in study groups

ADPKD patients had similar BUN, creatinine, estimated GFR, total cholesterol, HDL-cholesterol LDL-cholesterol and triglycerides levels as compared with control subjects (Table 1). ADPKD patients had significantly higher levels for hs-CRP [0.42 (0.22–1.19) vs. 0.31 (0.13–0.9) mg/dL, p < 0.001] and uric acid (4.8 ± 1.5 vs. 3.2 ± 0.9 mg/dL, p < 0.001) levels as compared with control subjects (Table 1).

### Echocardiographic and ultrasonographic findings in study groups

ADPKD patients had significantly higher levels for LVEF [72 (60–82) vs. 70 (65–82) %, p = 0.009], LVM (188 ± 55 vs. 154 ± 36 g, p < 0.001), CIMT [0.7 (0.4–1.2) vs. 0.5 (0.4–0.8) mm, p < 0.001], and EATT (6.8 ± 2.7 vs. 4.8 ± 1.2 mm, p < 0.001) as compared with control subjects (Table 1).

### Correlation between EATT and CIMT

**Fig. 1:**
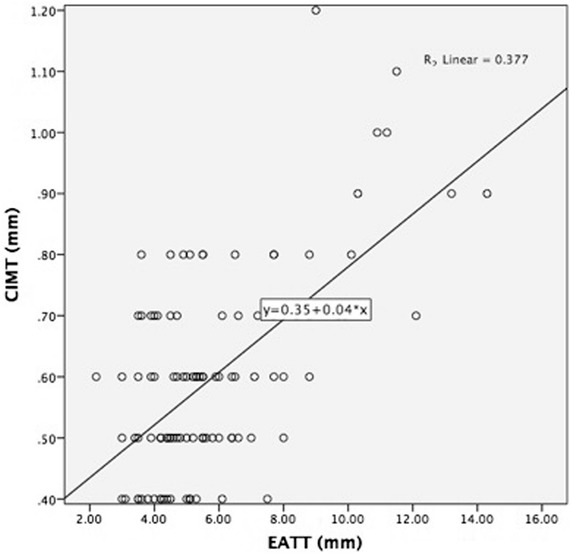
Correlation between epicardial adipose tissue thickness (EATT) and carotid intima-media thickness (CIMT) in patients with ADPKD

There was a significant positive correlation between EATT and CIMT (r = 0.58, p < 0.001) (Fig. 1).

### Multivariate linear regression analysis for factors predicting EATT and CIMT

Variables correlated in the univariate models with EATT were listed in Table 2. Multivariate linear regression analysis revealed independent correlates of EATT as having ADPKD (OR 10.2, 95 % CI 2.53–17.86, p = 0.01) and hs-CRP (OR 54.7, 95 % CI 37.44–72.01, p < 0.001). A higher age tended to be independently associated with a higher EATT (p = 0.06). Independent correlates of CIMT were increased EATT (OR 0.02, 95 % CI 0.01–0.04, p = 0.006), having ADPKD (OR 0.86, 95 % CI 0.2–1.6, p = 0.02), older age (OR 0.04, 95 % CI 0.004–0.07, p = 0.03) and lower HDL-cholesterol (OR −0.03, 95 % CI [−0.06] to [−0.004], p = 0.03).

**Table 2:** Multivariate linear regression analysis for factors predicting EATT

	Univariate correlation		Multivariate linear regression	
	r	p	OR	p
Age	0.41	<0.001	0.35 ([− 0.02] to [0.71])	0.06
ADPKD	0.417	<0.001	10.2 ([2.53] to [17.86])	*0.01*
GFR	−0.316	0.001	−0.17 ([− 0.39] to [0.04])	0.11
BMI	0.29	0.002	0.87 ([− 0.2] to [1.94])	0.11
Systolic BP	0.21	0.034	0.15 ([− 0.21] to [0.51])	0.4
hs-CRP	0.512	<0.001	54.7 ([37.44] to [72.01])	<*0.001*
Uric acid	0.477	<0.001	0.05 ([− 2.85] to [2.96])	0.9
LDL-C	0.216	0.027	−0.01 ([− 0.13] to [0.11])	0.8

Dependent variable: epicardial adipose tissue thickness (EATT)

*ADPKD* autosomal dominant polycystic kidney disease, *BMI* body mass index, *BP* blood pressure, *GFR* glomerular filtration rate, *hs*-*CRP* high sensitive C-reactive protein, *LDL* low density lipoprotein

The results in italics identify the statistically significant values

**Fig. 2:**
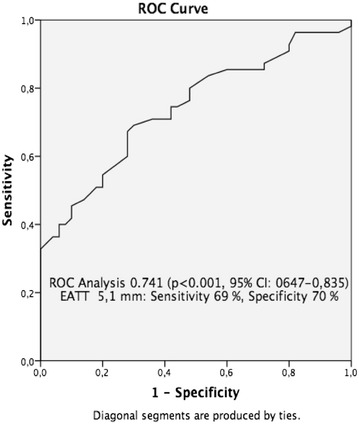
Receiver operating characteristic (ROC) curve of epicardial adipose tissue thickness (EATT) measured by echocardiography for predicting ADPKD

Receiver operating characteristics curve analysis was performed to describe optimal cut-off level for EATT with optimal sensitivity and specificity to distinguish patients with ADPKD and controls. Receiver operator characteristics curve (AUC under the curve 0.74, p < 0.001) analysis showed that an EATT of 51.5 mm or higher was 69 % sensitive and 70 % specific to distinguish between patients with ADPKD and controls (Fig. 2).

## Discussion

Our findings in a cohort of normotensive ADPKD patients with well-preserved renal function revealed higher levels for LVEF, LVM and serum uric acid levels in patient than in controls. Additionally, hs-CRP, CIMT and EATT levels were higher in ADPKD patients as compared with controls. Besides a significant positive correlation between CIMT and EATT levels, presence of ADPKD was determined to be an independent predictor both increased EATT and CIMT levels, while increased EATT also predicted the increased CIMT levels.

As mentioned earlier, our ADPKD patients have higher levels of LVM, even though they have similar arterial BP levels compared to the control group. This finding is supported by several other studies, and development of LVH in normotensive ADPKD patients may have caused by several reasons including; renin-angiotensin-aldosterone system activation, adrenergic stimulation, endothelial dysfunction and diastolic dysfunction (Ecder and Schrier 2009; Martinez-Vea et al. 2000; Kocyigit et al. 2014; Pietrzak-Nowacka et al. 2012; Chapman et al. 1997). In addition, preserved LVEF points to the presence of diastolic dysfunction. However, we did not evaluate left ventricular diastolic function in the study.

Likewise, numerous clinical reports confirmed the association between the echocardiographic EATT and cardiovascular risk factors, both traditional and novel, as well as with atherosclerotic disease (Kocyigit et al. 2014; Polak et al. 2011; Iacobellis and Willens 2009; Iacobellis et al. 2003; Lang et al. 2005; Kankaanpaa et al. 2006). Hence, echocardiographic measurement of EATT has been considered amongst the markers of early atherosclerosis (Iacobellis et al. 2008), while shown to be associated with increased CIMT in patients with metabolic syndrome (MetS) (Sengul et al. 2011), correlated with CIMT and arterial stiffness better than waist circumference in hypertensive subjects (Natale et al. 2009), and the best independent predictor of CIMT in human immunodeficiency virus infection associated with MetS (Iacobellis et al. 2007).

Our findings are consistent with data from past studies indicating presence of increased CIMT among both hypertensive and normotensive patients with ADPKD compared with healthy individuals (Widlansky et al. 2003; Sag et al. 2015; Turkmen et al. 2008). Although, EATT was shown to be positively correlated with CIMT in several diseases (Altun et al. 2014; Natale et al. 2009; Kim et al. 2013), our findings indicate potential of EATT to predict early atherosclerosis as well CIMT in ADPKD patients with well-preserved renal function for the first time in the literature.

Accordingly, being considered amongst markers of ED and thus of early atherosclerosis, increased levels for CIMT and EATT in our ADPKD patients with well-preserved renal function emphasize that during ADPKD, subclinical organ damage starts earlier than renal impairment (Ecder and Schrier 2009; Turkmen et al. 2008; Peterson et al. 2013).

In this regard, our findings emphasize the potential value of echocardiographic EATT measurements in evaluating subclinical target organ damage, and thus the likelihood of implementing strategies to control unfavorable cardiovascular risk factors and the development of future CVD in patients with increased EATT (Ecder and Schrier 2009; Pei et al. 2009).

Besides, on the basis of the documented correlation of eGFR to CRP in patients with CKD (Tong et al. 2007; Dubin et al. 2011), increased hs-CRP levels in our ADPKD patients with preserved renal function seems to indicate the likelihood of both CRP mediated inflammatory changes to occur at much earlier stages of renal disease. Also, positive correlation between EATT and hs-CRP emphasize the independent association of epicardial fat with inflammatory markers (Altun et al. 2014; Malavazos et al. 2008).

On the basis of several advantages such as simplicity, low cost, reliability, easy accessibility, rapid applicability, and good reproducibility echocardiographic EATT measurement has been suggested to offer a routine way of evaluating atherosclerotic and cardiovascular risk in a clinical setting with likely correlation with cardiometabolic risk factors and potential relevance in CAD and atherosclerotic vascular disease (Iacobellis and Willens 2009). Even though, CIMT is a well-known marker of atherosclerosis, EATT measurement is easier than CIMT measurement in the real world. Since, an extra probe is required for CIMT measurement, EATT might become preferable. In this regard our findings indicate EATT may serve as a simple tool and an independent predictor of subclinical atherosclerosis in ADPKD patients with preserved renal function.

Certain limitations to this study should be considered. First, small sample size might be disadvantage in achieving the statistical significance about the independent correlates of EATT and CIMT as well as to apply results of the present regression models to the entire population. Secondly, with the exception of hs-CRP, our study was lacking other oxidative stress markers. Thirdly, in our study, we only include patients with normotensive ADPKD with normal renal function, and excluded any other form of ADPKD. Another limitation is lack of long-term follow-up of patients in terms of cardiovascular events. Finally, the cross-sectional design made it impossible to establish any cause and effect relationships.

In conclusion, our findings indicate presence of early atherosclerotic changes in ADPKD patients with well-preserved renal function based on identification of increased EATT and CIMT. Our findings suggest that EATT, being simply measured by echocardiography and correlated with CIMT, can be used as a first-line measurement to detect early atherosclerosis in normotensive ADPKD patients. Further studies in large randomized populations are needed to improve our understanding pathophysiological mechanisms involved in early onset of ED in ADPKD patients with more robust and convincing evidence regarding the diagnostic and predictive properties of echocardiographic EATT to be considered as a routine method of evaluating cardiovascular risk in a clinical setting.
